# Clinical and patient‐reported outcomes of SPARE – a randomised feasibility study of selective bladder preservation versus radical cystectomy

**DOI:** 10.1111/bju.13900

**Published:** 2017-05-29

**Authors:** Robert A. Huddart, Alison Birtle, Lauren Maynard, Mark Beresford, Jane Blazeby, Jenny Donovan, John D. Kelly, Tony Kirkbank, Duncan B. McLaren, Graham Mead, Clare Moynihan, Raj Persad, Christopher Scrase, Rebecca Lewis, Emma Hall

**Affiliations:** ^1^ The Institute of Cancer Research London UK; ^2^ Royal Marsden NHS Foundation Trust London UK; ^3^ Royal Preston Hospital Preston and University of Manchester Manchester UK; ^4^ Royal United Hospital Bath Bath UK; ^5^ University of Bristol Bristol UK; ^6^ University College London Hospital London UK; ^7^ Patient Representative Edinburgh UK; ^8^ Western General Hospital Edinburgh UK; ^9^ Southampton General Hospital Southampton UK; ^10^ North Bristol NHS Trust Bristol UK; ^11^ The Ipswich Hospital NHS Trust Ipswich UK

**Keywords:** muscle‐invasive bladder cancer, radical cystectomy, selective bladder preservation, radiotherapy, randomised controlled trial, #BladderCancer, #blcsm

## Abstract

**Objectives:**

To test the feasibility of a randomised trial in muscle‐invasive bladder cancer (MIBC) and compare outcomes in patients who receive neoadjuvant chemotherapy followed by radical cystectomy (RC) or selective bladder preservation (SBP), where definitive treatment [RC or radiotherapy (RT)] is determined by response to chemotherapy.

**Patients and Methods:**

SPARE is a multicentre randomised controlled trial comparing RC and SBP in patients with MIBC staged T2–3 N0 M0, fit for both treatment strategies and receiving three cycles of neoadjuvant chemotherapy. Patients were randomised between RC and SBP before a cystoscopy after cycle three of neoadjuvant chemotherapy. Patients with ≤T1 residual tumour received a fourth cycle of neoadjuvant chemotherapy in both groups, followed by radical RT in the SBP group and RC in in the RC group; non‐responders in both groups proceeded immediately to RC following cycle three. Feasibility study primary endpoints were accrual rate and compliance with assigned treatment strategy. The phase III trial was designed to demonstrate non‐inferiority of SBP in terms of overall survival (OS) in patients whose tumours responded to neoadjuvant chemotherapy. Secondary endpoints included patient‐reported quality of life, clinician assessed toxicity, loco‐regional recurrence‐free survival, and rate of salvage RC after SBP.

**Results:**

Trial recruitment was challenging and below the predefined target with 45 patients recruited in 30 months (25 RC; 20 SBP). Non‐compliance with assigned treatment strategy was frequent, six of the 25 patients (24%) randomised to RC received RT. Long‐term bladder preservation rate was 11/15 (73%) in those who received RT per protocol. OS survival was not significantly different between groups.

**Conclusions:**

Randomising patients with MIBC between RC and SBP based on response to neoadjuvant chemotherapy was not feasible in the UK health system. Strong clinician and patient preferences for treatments impacted willingness to undergo randomisation and acceptance of treatment allocation. Due to the few participants, firm conclusions about disease and toxicity outcomes cannot be drawn.

## Introduction

Achieving local disease control is a critical step in treating muscle‐invasive bladder cancer (MIBC). A common approach is surgical removal of the bladder and adjacent organs, i.e. radical cystectomy (RC). Despite being a successful approach to cancer control, this is a major operation, in an often unfit and/or elderly population. It requires formation of a urinary diversion and has substantial associated morbidity and mortality rates 1, 2.

Radical radiotherapy (RT) is an alternative to RC 3, 4. It preserves a functioning bladder and avoids the risks of major surgery, but does not achieve local control for all patients and, if unsuccessful, requires subsequent salvage RC, which can be challenging 5. The relative efficacy of RT and RC has been debated extensively but, as randomised data are lacking, comparisons have been largely based on retrospective series, where inherent biases can make interpretation difficult 4, 6, 7. UK bladder cancer treatment guidelines released in 2015 recommend that patients with MIBC are offered a choice of RC or RT with a radiosensitiser 8.

There also exists a paucity of comparative data on the effects of both treatment options on patients’ quality of life. RC has been found to have a substantial negative impact on health‐related quality of life in the first year postoperatively 4, whilst patients who have received RT experience greater gastrointestinal dysfunction 9.

Several groups have hypothesised that RT would be more attractive as a treatment option if it were possible to select patients with tumours most likely to respond. This would minimise the need for salvage RC by undertaking immediate RC for patients predicted to have less chance of cure with RT.

Neoadjuvant chemotherapy before radical treatment improves survival in MIBC 10, 11 and studies have suggested that tumours that respond to neoadjuvant chemotherapy may achieve higher rates of local control with RT than those which do not 11, 12. Using chemotherapy in this way to select patients for RT achieved high levels of long‐term bladder preservation and avoided the need for surgery in most patients 13, 14. To test the efficacy of this approach we planned a randomised trial, with an initial feasibility study to compare, after neoadjuvant chemotherapy, a selective bladder preservation (SBP) strategy with patients undergoing RC.

## Patients and Methods

### Study Design

**Figure 1:**
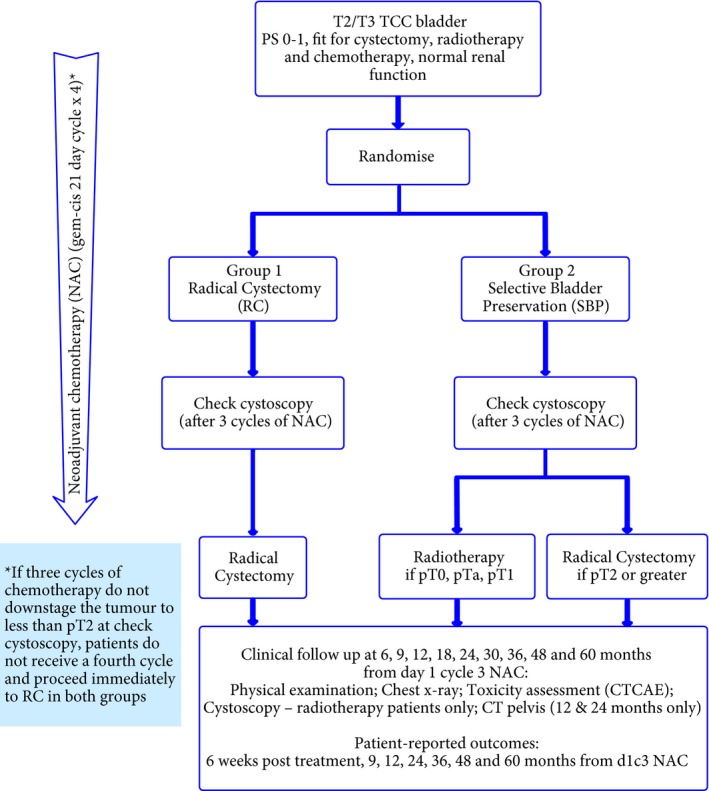
Trial schema.

SPARE (CRUK/07/011) was a multicentre phase III randomised controlled trial with an initial feasibility study (Fig. 1). The aims of the feasibility study were to determine viability of accrual for the phase III trial and assess compliance with the assigned treatment strategy. There was an embedded qualitative research programme, which has been previously reported 15, 16. The phase III trial was designed to determine if overall survival (OS) following bladder preservation is non‐inferior to that following RC for patients whose tumours respond to neoadjuvant chemotherapy.

Patients were recruited at UK NHS Trusts. All Trusts providing trial treatment had to provide details of surgical activity, including morbidity and mortality rates, for central review and confirmation of completion of a RT quality assurance programme prior to activation. Randomisation was by telephone to the Institute of Cancer Research, Clinical Trials and Statistics Unit (ICR‐CTSU). Participants were assigned 1:1 between SBP and RC using computer‐generated random permuted blocks (size 6 and 8), stratified by centre. Treatment allocation was not masked.

### Patients

Eligible patients provided written informed consent, were receiving neoadjuvant chemotherapy and fit for RT and RC, aged ≥18 years, had T2–T3 N0 M0 TCC of the bladder and WHO performance status of 0–1, with satisfactory haematological profile and kidney function. Key exclusion criteria were widespread carcinoma *in situ*; simultaneous upper tract, urethral or prostatic urethral TCC; untreated hydronephrosis; and invasive malignancy in the previous 5 years.

Initially treatment allocation took place during cycle two of neoadjuvant chemotherapy. Based on findings of the qualitative recruitment investigation 16, this timeframe was amended in August 2009 to allow randomisation at any time prior to a cystoscopy after chemotherapy cycle three (CT3) of neoadjuvant chemotherapy.

### Treatments

All patients received neoadjuvant chemotherapy. Gemcitabine (1 000 mg/m^2^ day 1 and day 8) and cisplatin (70 mg/m^2^) repeated every 21 days was recommended. All patients had a cystoscopy and tumour bed biopsy under general anaesthetic after CT3 of neoadjuvant chemotherapy, with subsequent treatment dependent on response.

Patients with ≥pT2 disease in both randomised groups proceeded immediately to RC within 6 weeks of CT3. Patients with histological downstaging (≤pT1), or a macroscopically normal bladder were classified as responders and received chemotherapy cycle 4 (CT4) of neoadjuvant chemotherapy with subsequent treatment determined by randomised allocation. Patients receiving RT were permitted to receive concomitant radiosensitising chemotherapy.

### SBP Group

Patients whose tumours responded to neoadjuvant chemotherapy started RT to the bladder 4–6 weeks after CT4. Two fractionation schedules in standard use in the UK were permitted (55 Gy/20 fractions or 64 Gy/32 fractions). The planning target volume was the bladder plus 1.5 cm margin, delivered by three‐dimensional conformal techniques.

### RC Group

Patients whose tumours responded to neoadjuvant chemotherapy received RC 4–6 weeks after CT4. RC consisted of resection of the bladder, prostate and seminal vesicles in men and bladder, uterus, ovaries and upper vagina in women. Pelvic lymphadenectomy, removing a minimum of 10 lymph nodes, was mandated and recommended to include dissection of the obturator nodes and external iliac nodes to the level of the iliac bifurcation and internal iliac nodes from the right and left side of the pelvis. The lateral limit of the dissection was the genito‐femoral nerve on the psoas muscle, and medial and posterior limits represented by the obturator nodes.

Orthotopic reconstruction using small or large bowel was encouraged; however, standard ileal conduit formation was also permitted.

### Trial Assessments

Before neoadjuvant chemotherapy, patients underwent physical examination, haematological and biochemical assessment, CT of the pelvis, chest X‐ray or CT, and maximal cystoscopic resection of tumour. Tumour control was assessed by physical examination, chest X‐ray and cystoscopy (if applicable) with follow‐up as shown in Figure 1. Adverse events were graded using Common Terminology Criteria for Adverse Events (CTCAE) version 3 17. Patient‐reported outcomes were collected, using paper European Organisation for the Research and Treatment of Cancer (EORTC) general cancer and MIBC modules [quality of life questionnaire – 30‐item core (QLQ‐C30) and 30‐item quality of life questionnaire for patients with muscle‐invasive bladder cancer (QLQ‐BLM30)] 18.

### Statistical Considerations

#### Endpoints

The primary endpoints of the feasibility study were accrual rate, bladder preservation rate in the SBP arm, and RC rate in the RC arm. For the phase III component, the primary endpoint was 5‐year survival. Secondary endpoints were treatment compliance, rate of salvage RC, toxicity, patient‐reported quality of life, and loco‐regional recurrence‐free survival, and metastasis‐free survival (MFS). For this analysis OS was treated as a secondary endpoint.

#### Sample size

The phase III trial was powered to evaluate non‐inferiority in the proportion of patients alive at 5 years between SBP and RC in those patients whose tumours responded to neoadjuvant chemotherapy. A 70% 5‐year survival was assumed 13, with the aim of excluding a decrease of ≥8% in the selective SBP group (corresponding to a critical hazard ratio for non‐inferiority of 1.34). Assuming an 80% neoadjuvant chemotherapy response rate, 1 015 patients would have been required to conclude non‐inferiority (80% power, one‐sided α = 0.05). For the phase III study to be considered feasible, it was recommended that 110 patients be randomised during the first 2 years; however, this was amended to 3 years, or a sustainable accrual rate of at least six patients per month, in August 2009 with the endorsement of the independent Trial Steering Committee 16. An analysis of the feasibility stage was planned to assess compliance with the SBP strategy, with the aim of excluding an initial bladder preservation rate of <60%. This stop/go criterion was based on a single‐arm phase II design and required 39/55 patients in the SBP arm to undergo RT following response to neoadjuvant chemotherapy to warrant continuation to phase III.

### Statistical Analysis

All randomised patients are included. The number of neoadjuvant chemotherapy responses was compared between groups using Fisher's exact test. Compliance with allocated treatment strategy was assessed by the proportion of patients: (i) with response after CT3 who received CT4 neoadjuvant chemotherapy and (ii) undergoing allocated treatment as their definitive treatment overall (i.e. RC in the RC arm, bladder preservation in SBP arm responders and RC in SBP arm non‐responders). In the SBP group, the bladder preservation rate was the proportion of patients who did not require RC after RT both overall and in the subset who received RT according to protocol guidelines, i.e. in the population who responded to chemotherapy and received CT4. Unless otherwise stated, proportions are presented with exact binomial 95% CIs.

Worst grade adverse events were compared by definitive treatment received and time to grade 3–4 event was estimated using Kaplan–Meier methods.

Time‐to‐event endpoints were assessed using Kaplan–Meier methods in the population of responders in both groups, and repeated according to both intention to treat (ITT) and definitive treatment received. Treatment effects were estimated using unadjusted Cox regression models, with a hazard ratio <1 indicating benefit for SBP in the ITT analysis or RT for the treatment received analysis. OS was defined as time to death from any cause; time to loco‐regional recurrence was calculated to first non‐muscle invasive bladder cancer (NMIBC) or MIBC recurrence in the bladder or recurrence in the pelvic nodes; MFS was time to the first of distant recurrence or death; disease‐specific survival was time to death following nodal or metastatic recurrence or unsalvageable local recurrence. All times are calculated from randomisation.

Quality‐of‐life data were analysed by treatment received and data were scored and missing data handled in accordance with the EORTC QLQ‐C30 scoring manual 19. For each QLQ‐C30 subscale, the mean change from baseline was calculated, with 99% CI, for each group at each time point and longitudinal plots of change from baseline were produced. Differences between groups in mean change from baseline to 12 months were assessed using analysis of covariance (ANCOVA), adjusting for baseline score.

Analyses are based on a snapshot of the database taken on 30th September 2014 and performed using Stata (Stata Statistical Software: release 13; StataCorp., College Station, TX, USA) 20.

### Research Governance

SPARE was funded by Cancer Research UK (CRUK/07/011, C1491/A9895). The study is registered (ISRCTN61126465), sponsored by the ICR, and approved by the South East Multicentre Research Ethics Committee. SPARE was managed by a multidisciplinary trial management group and overseen by Independent Data Monitoring (IDMC) and Trial Steering (TSC) committees.

## Results

### Patient Screening and Recruitment

The first patient was recruited on 20/07/2007 and the trial closed to recruitment on 12/02/2010 with 45 patients accrued on the advice of the IDMC and TSC due to failure to achieve target (stop/go) accrual rates.

Participating sites were requested to submit anonymised screening logs to the central coordinating centre on a regular basis throughout recruitment, to report patients with T2–3 N0 M0 bladder cancer who may be eligible for the trial. In all, 796 patients were reported, of whom 490 were ineligible, most of whom were not fit enough to receive all three SPARE treatment modalities (chemotherapy, RC and RT). A further 141 potentially eligible patients were not approached about participation, largely due to the complexity of the patient referral pathway, which meant that they were not identified as potentially eligible by the participating centre prior to radical treatment commencing 21.

**Figure 2:**
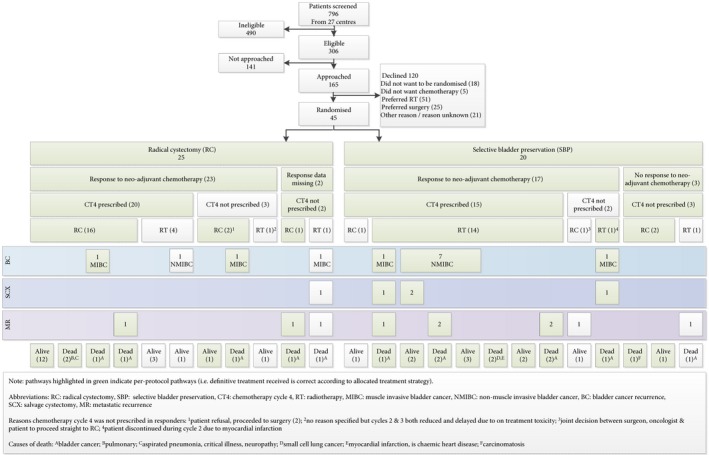
Patient flow through trial.

In all, 45/165 patients approached to participate consented, with 25 allocated to the RC group and 20 to SBP group. Of the 120 patients approached who declined, RT was preferred by 51 and RC by 25 (unknown 44) (Fig. 2).

### Baseline Characteristics and Compliance with Allocated Treatment

In all, 23/23 (100%) RC patients (two missing) and 17/20 (85%) SBP patients responded to neoadjuvant chemotherapy (*P* = 0.092) (Table 1). In all, 35 of these 40 patients with a response to neoadjuvant chemotherapy received CT4 in accordance with the protocol.

**Table 1:** Baseline characteristics and compliance with allocated treatment

Characteristic	RC	SBP (RT)	Total
Number of patients	**25**	**20**	**45**
Tumour characteristics			
**Gender, ** ***n*** **(%)**			
Male	22 (88)	18 (90)	40 (89)
Female	3 (12)	2 (10)	5 (11)
**Age**			
Mean (SD)	67.6 (6.1)	63.3 (8.5)	65.7 (7.5)
Median (min, max)	67 (58.2, 81.1)	63.2 (37.9, 75.2)	65.3 (37.9, 81.1)
Patient characteristics			
**Clinical stage, ** ***n*** **(%)**			
T2	22 (88)	14 (70)	36 (80)
T3a	0 (0)	2 (10)	2 (4)
T3b	1 (4)	2 (10)	3 (7)
Missing	2 (8)	2 (10)	4 (9)
**Pathological stage, ** ***n*** **(%)**			
pT1	1 (4)	1 (5)	2 (4)
pT2	24 (96)	19 (95)	43 (96)
**Tumour grade, ** ***n*** **(%)**			
G2	1 (4)	3 (15)	4 (9)
G3	24 (96)	17 (85)	41 (91)
**Type of chemotherapy, ** ***n*** **(%)**			
Gem‐Cis	24 (96)	20 (100)	44 (98)
Other (Gem‐Carbo)	1 (4)	0 (0)	1 (2)
Compliance with allocated treatment, *n* (%)*			
**Responder**	**23 (92)**	**17 (85)**	**40 (89)**
*RC*	*18 (72)*	*2 (10)*	*20 (44)*
*RT*	*5 (20)*	*15 (75)*	*20 (44)*
**Non‐responder**	**0 (0)**	**3 (15)**	**3 (7)**
*RC*	*– (–)*	*2 (10)*	*2 (4)*
*RT*	*– (–)*	*1 (5)*	*1 (2)*
**Response data missing**	**2 (8)**	**0 (0)**	**2 (4)**
*RC*	*1 (4)*	*– (–)*	*1 (2)*
*RT*	*1 (4)*	*– (–)*	*1 (2)*

Greyed cells indicate correct definitive treatment based on allocation and response to chemotherapy. *Figures in bold are numbers according to randomised groups, figures in italics indicate numbers according to treatment received.

Deviations from protocol defined treatment were frequent (Fig. 2). In all, 36/45 (80.0%, 95% CI 65.4–90.4%) received definitive treatment according to allocated group. Whilst, 19/25 (76%, 95% CI 54.9–90.6%) patients allocated RC underwent RC, with six (24%) receiving RT.

In the SBP group, 17/20 (85.0%, 95% CI 62.1–96.8%) received protocol defined treatment; 15/20 SBP patients (75%, 95% CI 50.9–91.3%) responded to neoadjuvant chemotherapy and received RT per protocol and two of the 20 patients (10%, 95% CI 1.2–31.7%) did not respond to chemotherapy and proceeded to RC per protocol. The other three patients were not treated in accordance with the SBP strategy: one non‐responder had RT after CT3 rather than proceeding to RC; two responded yet had RC (one after CT3 and one after CT4 of neoadjuvant chemotherapy).

In all, 22 participants overall (16 SBP; six RC) received RT, two with concomitant radiosensitisation. Five of the 22 (22.7%; 95% CI 7.8–45.4%) RT recipients subsequently underwent salvage RC, all due to recurrent bladder cancer (three MIBC, two NMIBC). The long‐term bladder preservation rate in the SBP group was 12/20 (60%) and was 11/15 (73%) in those SBP patients who received RT per protocol.

### Toxicity

**Figure 3:**
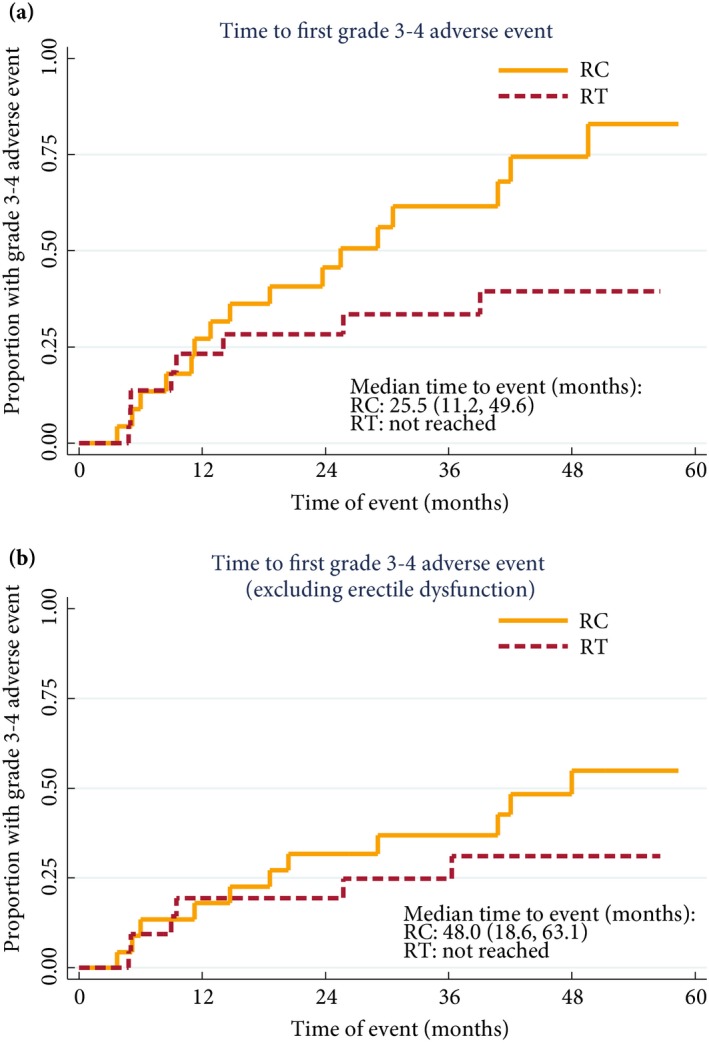
(**a**) Time to first CTCAE grade 3–4 toxicity by definitive treatment received and (**b**) when excluding erectile dysfunction.

More patients undergoing RC had CTCAE grade 3–4 toxicity [16/23 (70%) for RC; eight of 22 (36%) for RT; *P* = 0.038; which was 12/23 (52%) and six of 22 (27%) respectively, if erectile dysfunction is excluded] (Table 2, Fig. 3). The most common CTCAE grade 1–4 toxicity in patients undergoing RC was fatigue [15/23 (65%)]; and in patients receiving RT was fatigue and nocturia [both 12/22 (55%)].

**Table 2:** Worst overall toxicity grade by treatment received for all patients

CTCAE v3 grade	RC, *N* (%) (*N* = 23)	SBP (RT), *N* (%) (*N* = 22)	Total, *N* (%) (*N* = 45)
**All**			
0	0 (0)	0 (0)	0 (0)
1	2 (9)	6 (27)	8 (18)
2	5 (22)	8 (36)	13 (29)
3	10 (43)	8 (36)	18 (40)
4	6 (26)	0 (0)	6 (13)
Total grade 0–2	7 (30)	14 (64)	21 (47)
Total grade 3–4*	16 (70)	8 (36)	24 (53)
**Excluding ED**			
0	0 (0)	0 (0)	0 (0)
1	4 (17)	7 (30)	11 (48)
2	7 (30)	9 (39)	16 (70)
3	6 (26)	6 (26)	12 (52)
4	6 (26)	0 (0)	6 (26)
Total grade 0–2	11 (48)	16 (70)	27 (117)
Total grade 3–4†	12 (52)	6 (26)	18 (78)

ED, erectile dysfunction. *Two‐sided Fisher's exact test comparing number grade 3–4 events between the two groups *P* = 0.038. ^†^Two‐sided Fisher's exact test comparing number grade 3–4 events between the two groups *P* = 0.130.

### Cancer Control and Survival

**Figure 4:**
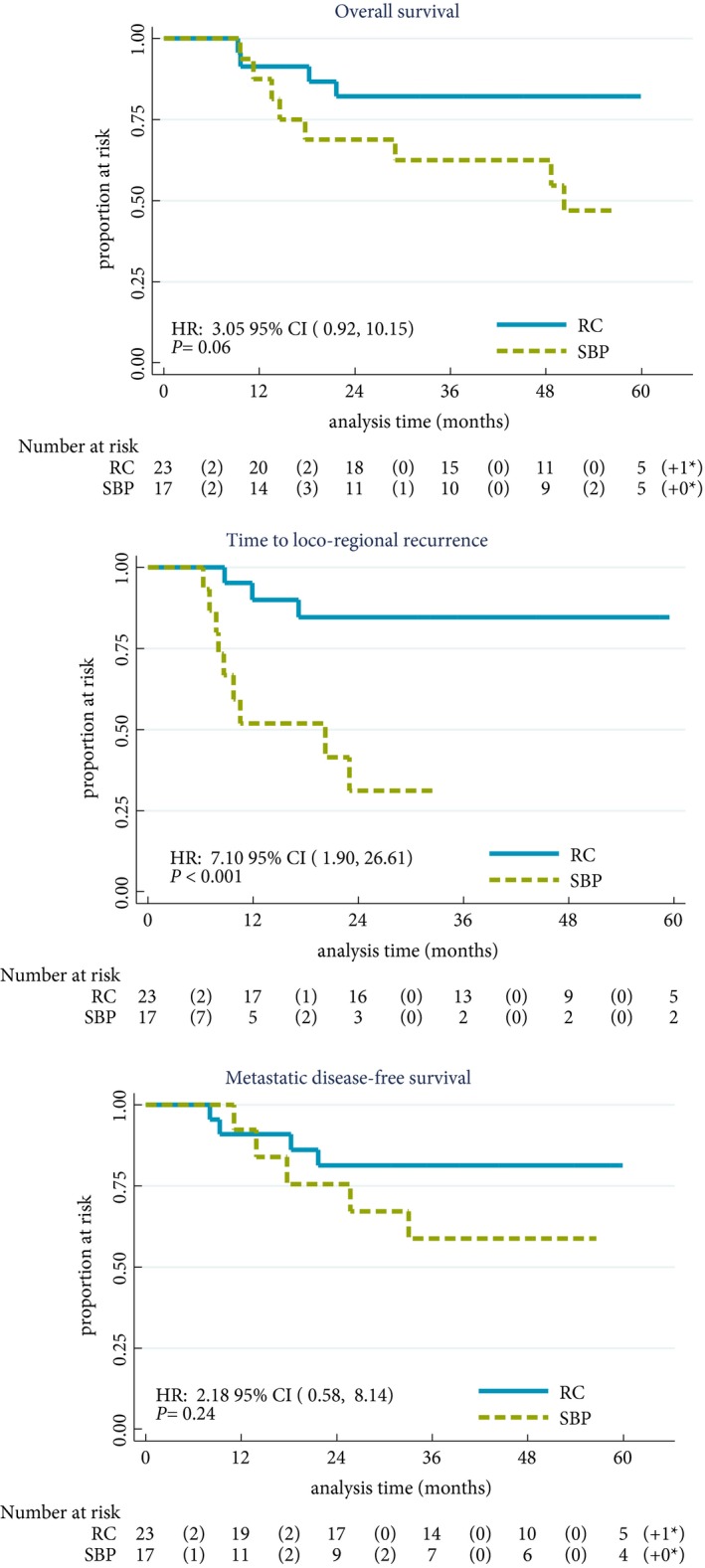
Time‐to‐event endpoints. Presented by allocated treatment for the population of patients who responded to chemotherapy. In all cases, patients with a second primary without a prior event were censored at the date of second primary and patients without an event were censored at the date last seen. Comparisons between groups were made using the log‐rank test.

The median (interquartile range) follow‐up was 58.0 (44.3–61.3) months. The hazard ratio for the randomised comparison of OS was 3.05 (95% CI 0.92–10.15; Fig. 4). Considering groups defined by definitive treatment received gave a hazard ratio of 1.83 (95% CI 0.55–6.07; Fig. S1). Given the wide CIs of the estimate, a survival difference between groups can be neither confirmed nor excluded and non‐inferiority cannot be claimed.

The loco‐regional recurrence rate at 2 years was lower in patients randomised to RC at 15.3% (95% CI 5.2–40.5%) vs 68.9% (95% CI 42.5–91.5%) in the SBP group (Fig. 4). Seven patients in the SBP group developed NMIBC recurrence, of whom five are long‐term survivors after salvage treatment. There was no evidence of difference in MFS (Fig. 4) or disease‐specific survival between randomised groups.

### Quality of Life

**Figure 5:**
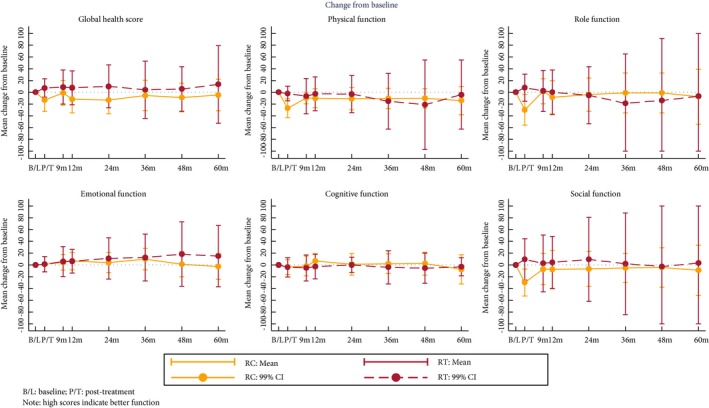
Mean change from baseline in EORTC QLQ‐C30 subscales.

**Figure 6:**
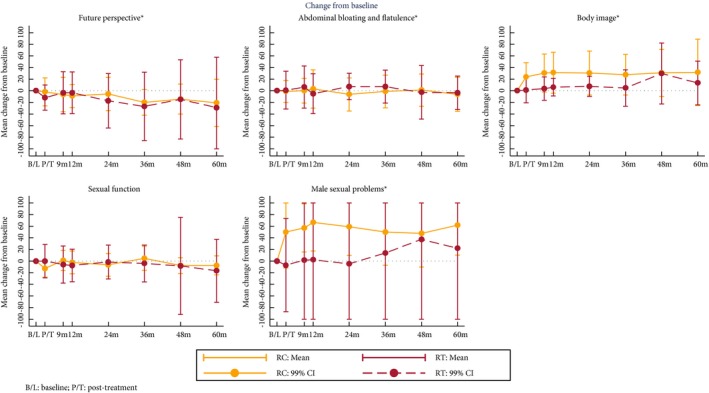
Mean change from baseline in EORTC QLQ‐BLM30 subscales.

Baseline subscale scores were similar between the groups. After 12 months, patients who received RT showed improvement in mean global health status and social functioning, whilst these declined in the RC group (Table 3). However, the confidence limits of the estimates of differences between groups are wide (Fig. 5). Changes over time in the QLQ‐BLM30 single items scores suggest a decline in body image and male sexual problems after RC that is less evident in RT patients (Fig. 6). With both treatments there is an improvement in future perspectives with time.

**Table 3:** Change in EORTC QLQ C30 subscale scores from baseline to month 12

	RC			SBP (RT)			RC vs SBP (RT)	
	*N*	Mean change from baseline	99% CI	*N*	Mean change from baseline	99% CI	Difference* (SBP – RC)	99% CI
Global health status	18	−11.6	−31.9 to 8.7	12	7.64	−11.9 to 27.1	14.2	−11.1 to 39.5
Physical function	18	−10	−23.9 to 3.9	10	−2.67	−22.9 to 17.6	7.76	−13.9 to 29.4
Role function	18	−8.3	−32.8 to 16.1	12	0	−25.5 to 25.5	15.27	−16.3 to 46.9
Emotional function	18	6.5	−6.4 to 19.4	12	6.25	−7.3 to 19.8	3.03	−11.9 to 18.0
Cognitive function	18	6.5	−4.7 to 17.6	12	−2.78	−16.8 to 11.2	−2.59	−14.5 to 9.3
Social function	18	−7.4	−35.1 to 20.3	12	4.17	−25.8 to 34.1	16.62	−16.6 to 49.9
Fatigue	16	−4.9	−28.4 to 18.7	12	−11.11	−35.5 to 13.3	−9.28	−33.2 to 14.7
Nausea/vomiting	18	−7.4	−17.2 to 2.3	12	0	−14.3 to 14.3	5.46	−8.3 to 19.2
Pain	18	1.9	−13.7 to 17.4	11	0	−14.2 to 14.2	−2.71	−19.4 to 14.0
Dyspnoea	18	0	−17.5 to 17.5	11	−3.03	−12.6 to 6.6	−3.47	−23.7 to 16.8
Insomnia	18	−5.6	−34.0 to 22.9	12	−2.78	−26.5 to 20.9	−7.72	−38.0 to 22.5
Appetite loss	18	0	−13.5 to 13.5	12	−2.78	−26.5 to 20.9	−3.53	−23.5 to 16.4
Constipation	18	−7.4	−30.3 to 15.4	12	2.78	−29.6 to 35.2	−2.86	−32.5 to 26.8
Diarrhoea	18	0	−7.8 to 7.8	12	0	−12.7 to 12.7	−0.69	−11.2 to 9.8
Financial problems	18	−3.7	−14.4 to 7.0	12	0	0 to 0	1.68	−10.8 to 14.1

CIs were constructed using Student's *t*‐distribution. No *P* values were calculated. High scores indicate better function for functional subscales, and high scores indicate worse symptoms/more problems for all other scales. *ANCOVA difference in the change in 12‐month subscale score from baseline between patients receiving SBP (RT) and patients receiving RC as definitive treatment, adjusting for baseline subscale score.

## Discussion

SPARE closed due to failure to meet the predefined minimum target recruitment rate, even though there had been extensive efforts and qualitative research to support recruitment 15, 16, 21.

One key criterion for assessing feasibility of phase III was to demonstrate acceptability of the randomised treatment strategies and viability of use of chemotherapy response to select patients for RT. At least 60% of those in the SBP group were anticipated to receive RT per protocol. Whilst an initial bladder preservation rate of 75% was seen in those receiving RT per protocol in the SBP arm, the small number of patients recruited resulted in wide CIs spanning 60%, such that the threshold to warrant continuation to phase III was not met. A 90% RC rate was anticipated in the RC group but RC was only performed in 76% of patients in this group.

Low randomisation rates and frequent deviations from allocated treatment suggest patients have a reluctance to allow randomisation to determine which of two contrasting treatment strategies they should receive. In accordance with the principles of good clinical practice, patients were made aware before randomisation that they could change their mind about participation in the trial at any stage without affecting the level of care they would receive. However, they were asked not to join the trial unless they thought they would be willing for their treatment to be determined by the SPARE protocol. Despite this request, a high proportion of treatment deviations, largely driven by patient choice, were observed.

An additional contributor to early closure of the study was the smaller than anticipated number of patients eligible for all treatment modalities. This, in addition to a lack of equipoise amongst clinicians 16, had a major impact. Undoubtedly, a proportion of patients approached for the present study showed an appetite for bladder preservation; many selecting RT when declining randomisation and a substantial proportion of participants receiving RT when not mandated by the protocol. This suggests that patients’ wishes for bladder preservation should be considered when discussing treatment options.

Robust conclusions cannot be made due to the limited sample size and are further complicated by poor compliance with assigned treatment strategy and differences in neoadjuvant chemotherapy response rates between the two randomised groups. Overall response to neoadjuvant chemotherapy was consistent with pilot work 14 and was higher than the pathological complete response rates in RC specimens reported in trials of neoadjuvant chemotherapy followed by RC 10, 22. This would suggest that cystoscopic examination under stages some patients and supports the rationale for recommending additional treatment even for a clinically normal‐looking bladder.

Loco‐regional recurrence‐free survival was worse after RT, mainly due to the incidence of NMIBC, which was more frequent than invasive recurrence. This is reported in other bladder preserving series 14, 23, 24 and suggests the bladder remains at high risk of developing second primary disease. This may indicate a role for preventative therapy such as that undertaken for NMIBC. Many cases of NMIBC can be salvaged with local treatment, thus the bladder preservation rate remained high as reported elsewhere 14, 24.

When comparing radical RT to RC, considering the frequency of ‘non‐salvageable’ recurrences may be more appropriate than overall recurrence rates. In the present study, the rate of non‐salvageable recurrences in responders to neoadjuvant chemotherapy is similar for RC (four of 23) and SBP (five of 17), as are OS and MFS. Observations from the present randomised trial are consistent with the results of population‐based studies 7, 25, 26, 27, non‐randomised single‐institution studies 6, and cross study comparisons 4, showing little evidence of inferior survival after SBP when compared to RC. A recent review of chemo‐RT studies for MIBC reported bladder cancer‐specific survival and OS rates of 50–82% and 36–74%, respectively 28; similar to those seen in like‐for‐like RC series. If, as our present results suggest, RT has less impact on quality of life than RC, this would provide additional rationale for consideration of bladder‐sparing therapy.

Few RT recipients had concomitant chemo‐RT, which has since been shown to significantly improve clinical outcomes 23. The technical delivery of RT has also improved with the advent of adaptive and image‐guided techniques 29, 30, 31, so one may expect improved outcomes with RT in the future. Likewise developments in surgery with increasing use of bladder reconstruction, enhanced recovery pathways 32, and minimally invasive techniques 33, 34, should result in benefits for patients.

The poor outcome of patients, whose tumours did not respond to neoadjuvant chemotherapy, whether or not they underwent RC, remains a concern and has been seen in other studies 14. Alternative systemic or palliative treatment options should perhaps be explored in this population.

Identification of predictive markers to help select patients for whom organ preservation may be a suitable option remains important. Recent work suggests that bladder cancer may consist of a variety of genetic sub‐types. It would be of interest to understand if certain of these subtypes are more or less likely to respond to chemotherapy or RT 35. Alternative candidates may be markers of DNA repair, with recently published work on MRE11 (meiotic recombination 11 homolog) and TIP60 (tat‐interactive protein) showing promising initial results 36, 37. These markers will need to be validated and then tested prospectively. Given experiences in SPARE, design of any such study will need to consider the powerful influence of patient and clinician preferences and issues of equipoise.

## Conclusions

A randomised phase III trial comparing SBP and RC after neoadjuvant chemotherapy was not feasible. Due to the small number of patients, firm conclusions about disease and toxicity outcomes following these interventions cannot be drawn, although high rates of bladder preservation appear to be achievable in chemotherapy responders without compromising OS.

## Conflicts of Interest

None.

**Abbreviations**
(N)MIBC: (non‐)muscle‐invasive bladder cancer
CT3(4): chemotherapy cycle three (four)
CTCAE: Common Terminology Criteria for Adverse Events
EORTC (QLQ‐C30) (QLQ‐BLM30): European Organisation for the Research and Treatment of Cancer (quality of life questionnaire – 30‐item core) (30‐item quality of life questionnaire for patients with muscle‐invasive bladder cancer)
ICR(‐CTSU): Institute of Cancer Research (Clinical Trials and Statistics Unit)
IDMC: Independent Data Monitoring Committee
ITT: intention to treat
MFS: metastasis‐free survival
OS: overall survival
RC: radical cystectomy
TSC: Trial Steering Committee

## Supporting information


**Figure S1.** Time‐to‐event endpoints. Patients who responded to chemotherapy by treatment received OS.
**Appendix S1** The SPARE centres.
