# Supplementary material for: Clinical and patient‐reported outcomes of SPARE – a randomised feasibility study of selective bladder preservation versus radical cystectomy
Source: BJU Int. 2017 May 29;120(5):639–50. doi: 10.1111/bju.13900 (PMC5655733; doi:10.1111/bju.13900)
Supplement: Supplementary file 1 — Figure S1. Time‐to‐event endpoints. Patients who responded to chemotherapy by treatment received OS. Appendix S1 The SPARE centres. [file BJU-120-639-s001.docx]

Supporting Information

Figure S1 Time to event endpoints

Patients who responded to chemotherapy by treatment received

OS

*events occurred after 60 months

LRR

MFS

*events occurred after 60 months

The SPARE centres

Principal and main co-investigators according to centre (number of patients recruited in bold).

† = SPARE Trial Management Group member

Bristol Haematology and Oncology Centre, **5**, Dr Amit Bahl, Dr Mark Beresford; Charing Cross Hospital, **4**, Dr Alison Falconer; Clatterbridge Cancer Centre, **1**, Dr Isabel Syndikus; Halton Hospital, Runcorn, **1**, Dr Isabel Syndikus; Huddersfield Royal Infirmary, **2**, Dr Ursula Hofmann; Lincoln County Hospital, **1**, Dr Thiagarajan Sreenivasan; Nottingham City Hospital, **2**, Dr Santhanam Sundar, Queen Elizabeth Hospital, Birmingham, **1**, Dr Anjali Zarkar, Dr Daniel Ford; Royal Marsden Hospital, London, **1**, Dr Vincent Khoo; Royal Marsden Hospital, Sutton, **9**, Prof Robert Huddart^†^; Royal Preston Hospital, **8**, Dr Alison Birtle^†^, Dr Omi Parikh; Sandwell General Hospital, West Midlands, **1**, Dr Ahmed El-Modir; Southampton General Hospital, **4**, Dr Catherine Heath, Dr Benjamin Mead; Southend General Hospital, **3**, Dr David Tsang; St James’s University Hospital, Leeds, **2**, Mr Alan Paul, Dr John Chester
